# Factors associated with an increase in alcohol consumption among users of online social networks in Russia in the early months of the COVID-19 pandemic

**DOI:** 10.1192/j.eurpsy.2022.788

**Published:** 2022-09-01

**Authors:** A. Gil

**Affiliations:** I.M. Sechenov First Moscow State Medical University (Sechenov University), Higher School Of Health Administration, Moscow, Russian Federation

**Keywords:** social networks, Russia, alcohol, Covid-19

## Abstract

**Introduction:**

The various restrictions and physical distancing introduced inRussia in early months of COVID-19pandemic could have a particular impact on people who use alcohol and create new needs for prevention and treatment of associated disorders.

**Objectives:**

The study was aimed at assessing changes in alcohol consumption among users of online social networks in Russia.

**Methods:**

During June-September 2020,1518 adult users (18+) of the most popular social networks in Russia (Odnoklassniki, VKontakte,Facebook, Twitter), completed an anonymous online survey regarding alcohol use changes in the first months of COVID-19 pandemic. Binary logistic regression was used to estimate associations of increased alcohol consumption with socio-demographic and pandemic-related factors.

**Results:**

35.4%of men and 25.6%of women increased their frequency of drinking; 24.9%of men and 17.7%of women increased their typical one-time volume of alcohol consumption,and 28.5% of men and 27.9% of women increased frequency of heavy episodic drinking in the early months of pandemic. After mutual adjustment of independent variables,age18-29 years(OR=1.710;95%CI 1.002-2.917), very strong restrictions of everyday life(3.127; 1.011-9.675)and severe negative consequences in relation to professional or financial situation due to spread ofSARS-CoV-2 (2.247; 1.131-4.465) were positively associated with increase in frequency of drinking. The odds of increase in frequency of heavy episodic drinking were more than twice higher(2.329; 1.001-5.428) among those who experienced severe negative consequences to their professional/financial situation.Higher typical frequency,larger one-time volume of alcohol use,and higher frequency of heavy episodic drinking before pandemic were positively associated with increase of alcohol consumption in early months of pandemic.

**Conclusions:**

Timely monitoring of changes in alcohol consumption during pandemic can allow prevention of alcohol-related disorders, including mental disorders, among users of online social networks.

**Disclosure:**

No significant relationships.

